# A Novel HPLC Method for Direct Detection of Nitric Oxide Scavengers from Complex Plant Matrices and Its Application to *Aloysia triphylla* Leaves

**DOI:** 10.3390/molecules23071574

**Published:** 2018-06-28

**Authors:** Didier Fraisse, Alexandra Degerine-Roussel, Alexis Bred, Samba Fama Ndoye, Magali Vivier, Catherine Felgines, François Senejoux

**Affiliations:** 1 Université Clermont Auvergne, INRA, UNH, F-63000 Clermont-Ferrand, France; didier.fraisse@uca.fr (D.F.); alexandra.degerine@uca.fr (A.D.-R.); alexis.bred@uca.fr (A.B.); catherine.felgines@uca.fr (C.F.); 2 Laboratory of Organic and Therapeutic Chemistry, Faculty of Medicine, Pharmacy and Odontology (F.M.P.O.), Cheikh Anta Diop University (U.C.A.D.), Dakar-Fann PB 5005, Senegal; sambabathie0806@yahoo.fr; 3 UMR IMoST 1240 Inserm, Université Clermont Auvergne, 63005 Clermont-Ferrand, France; magali.vivier@uca.fr

**Keywords:** nitric oxide scavengers, pre-column HPLC method, *Aloysia triphylla*, phenolics, antioxidants

## Abstract

The present study aimed at developing an original pre-column HPLC assay allowing rapid characterization of nitric oxide (NO) scavengers from complex plant extracts. Sodium nitroprusside (SNP) was employed as a NO donor and spiked with an aqueous extract from *Aloysia triphylla* leaves prior to HPLC analysis. Relying on the ability of radical scavenging constituents to be oxidized upon reaction with radicals, this assay successfully allowed direct identification of three potential NO scavengers, including verbascoside, isoverbascoside, and luteolin-7-*O*-diglucuronide. These three phenolics were also individually assessed for their NO scavenging activities by using a Griess colorimetric assay. With respective IC_50_ values of 56 ± 4, 51 ± 3, and 69 ± 5 µg/mL, verbascoside, isoverbascoside, and luteolin-7-*O*-diglucuronide were all reported as potent NO scavenging compounds, confirming the efficiency of the SNP spiking HPLC assay. The present method can, thus, be considered as a valuable and effective approach for speeding up the discovery of NO scavenging constituents.

## 1. Introduction

Nitric oxide (NO) is an important signaling molecule with dual effects. Generated by nitric oxide synthases through the conversion of l-arginine to l-citrulline, NO plays a key role in regulating vasodilation, neurotransmission, and the immune system, as well as cardiovascular and renal functions [1,2,3]. Low concentrations are, in most cases, sufficient to exert these beneficial effects. However, overproduction of this free radical may induce several undesired deleterious effects, including inflammatory and autoimmune diseases [4,5]. The search for substances capable of preventing overproduction of NO has, therefore, received increasing attention, and numerous extracts of edible and/or medicinal plants have been shown to exert potent NO scavenging activities [6,7]. Nevertheless, the main pitfall to drug discovery or plant extract standardization remains the characterization of bioactive principles. Traditional bioassay-guided fractionation has been successfully conducted in some investigations [8], but several major limitations arise from this time-consuming, labor intensive, and expensive strategy. Thus, there is a need to develop new approaches capable of speeding up the discovery of NO scavenging natural compounds. Of interest, several pre-column HPLC spiking methods have been recently reported to directly detect radical scavenging compounds from complex matrices, such as plant extracts [9,10]. These experiments consist of spiking extracts with a radical solution prior to HPLC analysis, and rely on the ability of antioxidant constituents to be oxidized upon reaction with radicals. On this basis, peak areas of radical scavenging compounds are reduced in HPLC chromatograms of spiked extracts whereas peak areas of inactive constituents are not affected. Of note, such a kind of approach has already been successfully adapted to identify DPPH [11] and ABTS [12], as well as peroxynitrite scavengers [13]. However, a method capable of detecting NO scavengers has yet to be devised. By using sodium nitroprusside (SNP) as an NO source, the present study aimed at developing a novel spiking HPLC assay that allows direct detection of NO scavenging constituents from herbal products. Lemon verbena (*Aloysia triphylla* (L’Hér.) Britt., Verbenaceae), a widely consumed herbal tea known to exert significant anti-oxidant and anti-inflammatory effects [14,15], was chosen as the case study plant material.

## 2. Results and Discussion

### 2.1. NO Scavenging Activity and Total Phenolic Content of an Aqueous Extract from *A. triphylla* Leaves

Native to western South America, *A. triphylla* is a widely consumed edible plant whose leaves are mostly employed as herbal tea. To be relevant to this mode of consumption, an aqueous infusion was prepared. *A. triphylla* aqueous extract (ATAE) was first assessed for its NO scavenging activity by using common colorimetric evaluation. This method relies on the principle that, in aqueous solution and at physiological pH, SNP spontaneously generate NO radicals, which subsequently react with oxygen to produce nitrite ions that can be estimated with Griess reagent. Scavengers of NO compete with oxygen, thus, leading to a lower production of nitrite ions. The present study demonstrates, for the first time, that ATAE exerts potent NO scavenging effects, as attested by its low IC_50_ value of 231 ± 17 µg/mL. These results tend to suggest that *A. triphylla* leaves can be regarded as a suitable herbal product to counteract NO overproduction. Considering that polyphenols are regarded as the most prevalent radical scavenging phytochemicals in the plant kingdom [16], total phenolic content of the extract was also evaluated. Of interest, a substantial value of 148 ± 1 mg gallic acid equivalent per g of dried extract was determined for ATAE. Taken together, these preliminary results indicated that ATAE was a suitable candidate for the development of a straightforward HPLC method, aiming at directly detecting NO scavenging compounds from a complex plant extract. 

### 2.2. Separating Constituents from ATAE by HPLC

**Figure 1:**
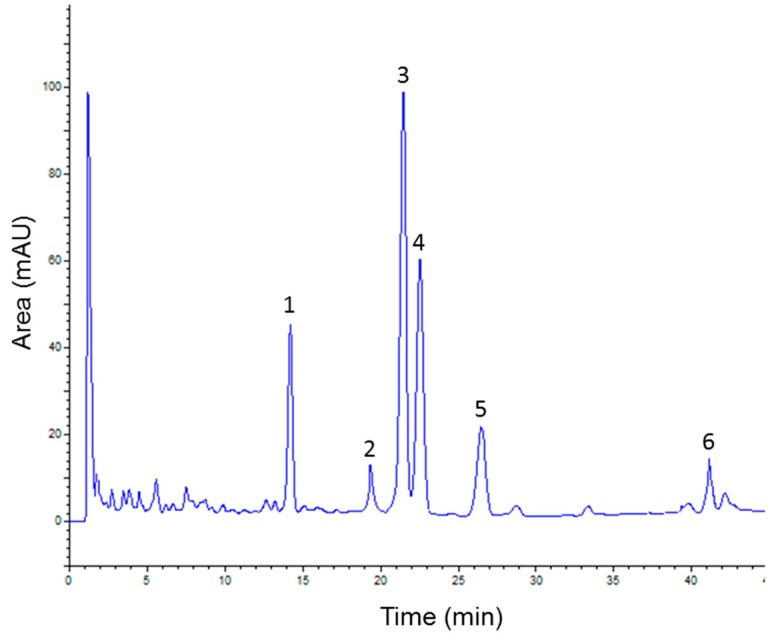
HPLC-UV profile of aqueous extract from *Aloysia triphylla* leaves with detection at 280 nm. Peaks: 1, luteolin-7-*O*-diglucuronide; 2, apigenin-7-*O*-diglucuronide; 3, verbascoside; 4, diosmetin-7-*O*-diglucuronide; 5, isoverbascoside; and 6, apigenin-7-*O*-glucoside.

Ensuring the separation of all compounds in a plant extract is a crucial first step in analytical HPLC. Different modes of elution and mobile phase compositions were screened to obtain chromatograms with a sufficient resolution and within an acceptable time of analysis. Under the chosen conditions, a satisfying resolution was achieved and all the major compounds reached base-line separation. As illustrated in Figure 1, six main constituents were observed on the ATAE chromatogram.

### 2.3. SNP Spiking HPLC Analysis for Screening of Main Scavengers in ATAE

**Figure 2:**
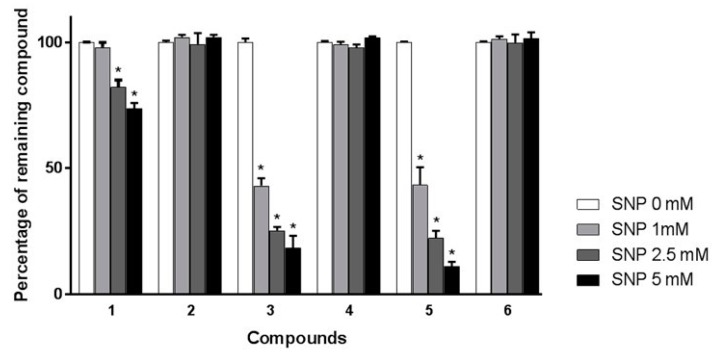
Percentage of remaining compounds (**1**–**6**) after incubation of aqueous extract from *Aloysia triphylla* leaves with various concentrations of SNP (0, 1, 2.5, and 5 mM). Data are presented as means ± SEM (*n* = 3). * *p* < 0.05 vs. control (SNP = 0 mM).

Unlike commonly employed synthetic radicals, such as DPPH and ABTS [10,12], NO is not stable enough to be prepared prior to spiking experiments. Thus, the present method had to be developed with a reagent able to extemporaneously generate NO during pretreatment of the extract. As for colorimetric evaluation, SNP was chosen as the NO donor. Spiking experiments were performed with three different concentrations of SNP (1, 2.5, and 5 mM). It was hypothesized that pretreatment of ATAE with a low concentrated solution of SNP would only oxidize the most reactive scavengers, while solutions containing higher concentrations of SNP would affect additional antioxidant constituents. As shown in Figure 2, the chromatogram of ATAE spiked with a 1 mM solution of SNP indicated that peak areas of compounds **3** and **5** were significantly reduced (*p* < 0.05) whereas other constituents were not affected. As expected, peak area diminutions were more pronounced when higher concentrations of SNP were employed (2.5 and 5 mM). Indeed, compound **1** was significantly affected by the SNP pretreatment at these two concentrations (*p* < 0.05). In addition, compounds **3** and **5** almost disappeared from the corresponding chromatograms. Conversely, the peak areas of compounds **2**, **4**, and **6** were not significantly modified over all the SNP concentrations. According to these spiking experiments, it was possible to assume that three constituents (**1**, **3**, and **5**) are mainly contributing to ATAE scavenging effect, while compounds **2**, **4**, and **6** only play a negligible role. To confirm this hypothesis and to validate the proposed method, all major constituents of the extract were identified and assessed for their individual NO scavenging activities.

### 2.4. Characterization and Identification of the Main Constituents of ATAE

Analysis of the UV data of the six major components of ATAE revealed the presence of two different classes of metabolites (Table 1). Indeed, compounds **3** and **5** showed highly similar profiles with two maximum absorption bands at 217 nm and 325–330 nm, which were consistent with caffeoyl derivatives [17]. On the other hand, with maximum absorption bands at 250–270 nm and 330–350 nm, the UV profiles of constituents **1**, **2**, **4**, and **6** were typical of flavonoid derivatives [18]. These assumptions were further confirmed by examining previous chemical investigations of *A. triphylla* [19] and by comparing retention times and UV spectra of ATAE components with that of authentic commercial standards. Compounds **2** and **6** were corresponding to flavonoid derivatives, and were identified as apigenin-7-*O*-diglucuronide and apigenin-7-*O*-glucoside, respectively. In addition, compounds **3** and **5** were characterized as two caffeoyl phenylethanoid glycosides, respectively known as verbascoside and isoverbascoside. However, compounds **1** and **4** did not match with any available standards and purification of these two constituents was achieved to unambiguously determine their structures. ATAE was first fractionated using gel filtration chromatography and two fractions containing important amounts of the targeted compounds were subsequently submitted to semi-preparative HPLC purification. ^1^H and ^13^C NMR analyses were then performed on the two purified compounds and confirmed their assumed flavonoid nature. Indeed, compound **1** exhibited characteristic signals of a luteolin glycoside, while the NMR data of compound **4** matched with a diosmetin derivative. In both cases, typical signals attributable to two β-glucuronide units were observed. Deeper investigations of NMR analyses allowed the unequivocal identification of compounds **1** and **4**, which were, respectively, characterized as luteolin-7-*O*-diglucuronide and diosmetin-7-*O*-diglucuronide. All spectral data of these compounds agreed with respective published data [20,21]. Of note, the occurrence of compounds **1**, **2**, **3**, **5**, and **6** is consistent with previous phytochemical investigations of *A. triphylla* leaves [19,20]. By contrast, diosmetin-7-*O*-diglucuronide (**4**) is unambiguously identified for the first time in this species. Also known as fargenin C, this compound is very scarcely distributed in the plant kingdom and had only been isolated once in *Meehania fargesii*, an Asian species belonging to the Lamiaceae family [21].

### 2.5. NO Radical Scavenging Activity of Isolated Components

To ascertain the efficiency of the present HPLC spiking assay, all the compounds affected by SNP pretreatment (**1**, **3**, and **5**) were individually assessed for their NO scavenging properties by using a common colorimetric method. In addition, two assumed inactive constituents (**4** and **6**) were also evaluated to be employed as negative controls. As indicated in Table 2, very low IC_50_ values were determined for compounds **1**, **3**, and **5**, confirming that a decrease in peak area during spiking experiments is indicative of NO scavenging properties. Of note, constituents **3** and **5** were both shown to exert higher NO scavenging activity than the positive control, ascorbic acid. The presumed weak effect of compounds **4** and **6** was also confirmed, as attested by their IC_50_ being superior to 200 µg/mL.

All these results are consistent with SNP spiking HPLC experiments, establishing that the method has successfully led to the detection of bioactive constituents of the studied extract. Additionally, compounds **3** and **5** were confirmed to be significantly more effective than compound **1** (*p* < 0.05), signifying that pretreatment with a low concentrated solution of SNP had only oxidized the most reactive scavengers. Of interest, the present study reports, for the first time, the potent NO scavenging properties of **1**, thus, highlighting the efficacy of the spiking method in the search of new NO scavengers. It can also be regarded as an effective experiment to quickly determine bioactive markers from plant extracts, and may be of major interest to support the standardization process of herbal products. Besides, it must be noted that this assay can be considered as more physiologically relevant than a DPPH spiking experiment. Indeed, in addition to using an endogenous radical, SNP spiking experiments are performed in aqueous solutions buffered at pH 7.4 while DPPH cannot be solved in water solution. It is well known that the pH value, as well as the solvent composition, strongly influences the reactivity of radical scavenging compounds [22].

## 3. Materials and Methods

### 3.1. Plant Material and Reagents

Dried leaves of *A. triphylla* were obtained from Biosphère 99 (Saint Bonnet de Rochefort, France). Acetonitrile was of chromatographic grade Carlo Erba Reagents SAS (Val de Reuil, France). Phosphoric acid (purity 85%) was purchased from VWR prolabo (Fontenay-sous-Bois, France). Sodium nitroprusside (SNP), *N*-(1Naphthyl) ethylenediamine dihydrochloride (NED), and sulfanilamide were bought from Sigma–Aldrich Chemical (Saint Quentin Fallavier, France). SNP solutions were freshly prepared in distilled water every half-day and kept protected from light.

### 3.2. Preparation of Aqueous Extract from *A. triphylla* Leaves

An infusion of *A. triphylla* leaves was prepared by adding 500 mL of boiling distilled water to powdered plant material (10 g) and was left at room temperature for 30 min. The extract was then filtered and concentrated under reduced pressure using a rotary evaporator. The obtained dried residue (2.57 g) was kept at 4 °C until further analyses.

### 3.3. Colorimetric NO Scavenging Assay and Total Phenolic Content Evaluation

Nitric oxide scavenging colorimetric assay was performed using Griess reagent as previously described by Silva and Soysa [23]. Briefly, SNP (10 mM in PBS) was mingled with different concentrations of ATAE (50–300 µg/mL in PBS) or pure compounds (5–200 µg/mL in PBS). A reaction mixture without extract or pure compound was also prepared on the same basis to be employed as the negative control. After 2 h incubation at 37 °C, Griess reagent (1% sulfanilamide, 5% phosphoric acid, and 0.1% NED) was added to the reaction mixtures. Absorbance was recorded at 540 nm after 10 min incubation. NO scavenging activities were expressed as IC_50_ values, which correspond to the concentration required to reduce 50% of the NO formation. Ascorbic acid was employed as the positive control.

Total phenolic content (TPC) was estimated by the Folin–Ciocalteu method as previously reported by Meda et al. [11]. The amount of TPC was indicated as a milligram of gallic acid equivalent per gram of dried extract.

### 3.4. HPLC Analysis of ATAE

Analytical HPLC consisted of two L7100 pumps, an L7200 autosampler, an L2450 LaChrom Elite diode array detector, a D7000 interface system controller, and an EZ Chrom Elite software (VWR-Hitachi, Radnor, Pennsylvania, PA, USA). ATAE samples (2 mg/mL) were analyzed using a LiChrospher^®^ RP8 column (125 × 4 mm, 5 µm particle size). A gradient elution was employed with a mobile phase consisting of water containing 1% of phosphoric acid (A) and acetonitrile (B). The program was set as follows: 0–15 min, 10–15% B; 15–25 min, 15% B; 25–40 min, 15–20% B; 40–50 min, 20–40% B; 50–60 min, 40–60% B. The flow rate was 1.0 mL/min and the injection volume was 30 µL. All analyses were performed at a detection wavelength of 280 nm and the column was maintained at ambient temperature.

### 3.5. Pre-Column SNP–HPLC Analysis for Screening of Main NO Scavengers in ATAE

Briefly, ATAE was dissolved in PBS at a concentration of 10 mg/mL and was subsequently mixed with SNP solutions of different concentrations (1, 2.5, and 5 mM, final concentrations) at the ratio of 1:5 (*v*/*v*). The resulting mixtures were incubated for 30 min at 37 °C prior to HPLC analyses in previously reported conditions (Section 3.4). As a control, ATAE was identically treated except that PBS was substituted for the SNP solution. All solutions were prepared and analyzed in triplicate. The percentage of the remaining compound was calculated as the ratio between the compound peak area after the reaction with SNP divided by the compound peak area of the control sample.

### 3.6. Isolation and Identification of Compounds ***1*** and ***4***

A portion of ATAE (500 mg) was subjected to exclusion chromatography (Sephadex^®^ LH-20, 75 g) eluted with H_2_O/MeOH (40:60) to yield a total of 8 combined fractions. Fractions 6 (30 mg) and 7 (40 mg) were then submitted to semi-preparative HPLC (Nucleosil^®^ RP-18, 250 mm × 100 mm, 5 µm particle size) eluted with a mobile phase consisting of water containing 0.1% trifluoroacetic acid and acetonitrile to obtain compounds **1** (7 mg) and **4** (11 mg). Proton and carbon nuclear magnetic resonance spectra were recorded in DMSO-*d*_6_ on a Bruker DRX 500 spectrometer (11.7 T:^1^H:500 MHz, ^13^C:125 MHz) (Bruker Biospin SAS, Wissembourg, France).

### 3.7. Statistical Analyses

The statistical significance of difference was analyzed by one-way ANOVA, followed by a Fisher’s LSD test, and values *p* < 0.05 were considered significant. All data are indicated as mean ± standard error of mean (SEM, *n* = 3).

## 4. Conclusions

In the present study, a novel SNP spiking HPLC assay has been successfully devised to quickly and directly detect NO scavengers from *A. triphylla* leaves. Three compounds, including luteolin-7-*O*-diglucuronide, as well as verbascoside and isoverbascoside, were shown to be the main contributors to the activity of the studied extract. It must be noted that the potent NO scavenging activities of luteolin-7-*O*-diglucuronide is reported for the first time, thus, highlighting the usefulness of this novel approach in the search of new bioactive chemical entities. Of interest, this method can be applied in all laboratories with common HPLC-UV equipment and can, thus, be widely adopted for the analysis of other NO scavenging plant extracts.

## Figures and Tables

**Table 1:** Retention time and UV maximum absorption of major constituents from *Aloysia triphylla* leaves.

Peak Number	Compound	Retention Time (min)	UV, *λ*_max_ (nm)
1	Luteolin-7-*O*-diglucuronide	14.3	255, 347
2	Apigenin-7-*O*-diglucuronide	19.3	265, 333
3	Verbascoside	21.6	217, 330
4	Diosmetin-7-*O*-diglucuronide	22.9	253, 346
5	Isoverbascoside	26.8	217, 326
6	Apigenin-7-*O*-glucoside	41.4	266, 332

**Table 2:** Nitric oxide scavenging activity of major constituents from *Aloysia triphylla* leaves.

Compound	Nitric oxide Scavenging Activity (IC_50_, µg/mL)
Luteolin-7-*O*-diglucuronide	69 ± 5 ^b^
Verbascoside	56 ± 4 ^a^
Diosmetin-7-*O*-diglucuronide	>200 ^c^^,^*
Isoverbascoside	51 ± 3 ^a^
Apigenin-7-*O*-glucoside	>200 ^c,^*
Ascorbic acid (positive control)	71 ± 2 ^b^

Values with different superscripts are significantly different (*p* < 0.05). * For statistical analysis, IC_50_ values of diosmetin-7-*O*-diglucuronide and apigenin-7-*O*-glucoside was assumed to be 200 µg/mL.

## Supplementary Materials

Supplementary File 1
