# Supplementary material for: A Novel HPLC Method for Direct Detection of Nitric Oxide Scavengers from Complex Plant Matrices and Its Application to Aloysia triphylla Leaves
Source: Molecules. 2018 Jun 28;23(7):1574. doi: 10.3390/molecules23071574 (PMC6100114; doi:10.3390/molecules23071574)
Supplement: Supplementary File 1 [file molecules-23-01574-s001.zip › molecules-318709-SI.pdf]

A novel HPLC method for direct detection of nitric oxide scavengers from complex plant matrices and its application to *Aloysia triphylla* leaves

Didier Fraisse [1], Alexandra Degerine-Roussel [1], Alexis Bred [1], Samba Fama Ndoye [2], Magali Vivier [3], Catherine Felgines [1], François Senejoux [1*]

[1] Université Clermont Auvergne, INRA, UNH, F-63000 Clermont-Ferrand, France
[2] Laboratory of Organic and Therapeutic Chemistry, Faculty of Medicine, Pharmacy and Odontology (F.M.P.O.), Cheikh Anta Diop University (U.C.A.D.). PB 5005 Dakar-Fann, Sénégal.
[3] UMR IMoST 1240 Inserm, Université Clermont Auvergne, 63005 Clermont-Ferrand, France
* Correspondence: francois.senejoux@uca.fr, Tel.: +33 4 73 17 80 33, fax number: +33 4 73 17 80 37

List of Supporting Information

Figure S1: ^{1}H NMR spectrum of **4** in DMSO-d6 (500 MHz).

Figure S2: ^{13}C NMR spectrum of **4** in DMSO-d6 (125 MHz).

Figure S3: UV spectra of identified compounds (1-6).

Figure S1: ^{1}H NMR spectrum of **4** in DMSO-d6 (500 MHz).

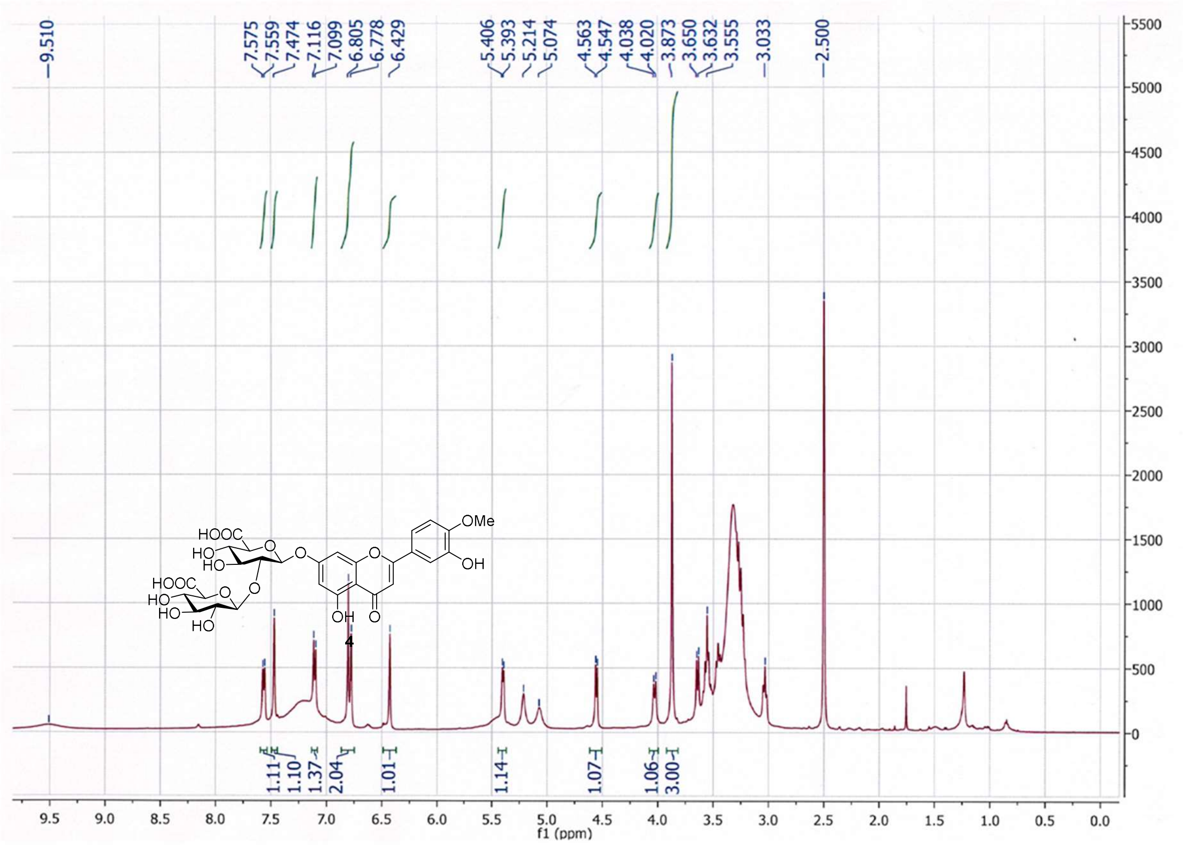

Figure S2: 13C NMR spectrum of **4** in DMSO-d6 (125 MHz).

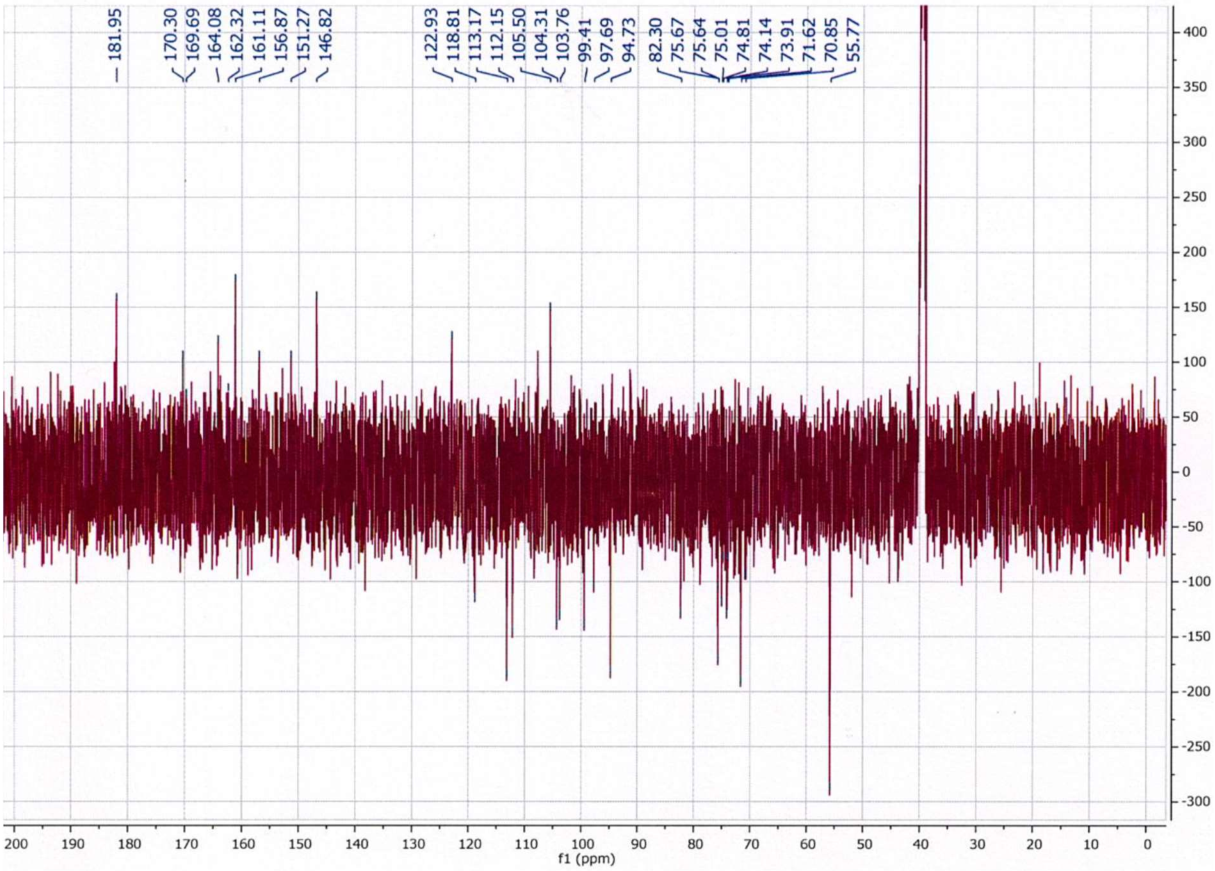

Figure S3: UV spectra of identified compounds (1-6).

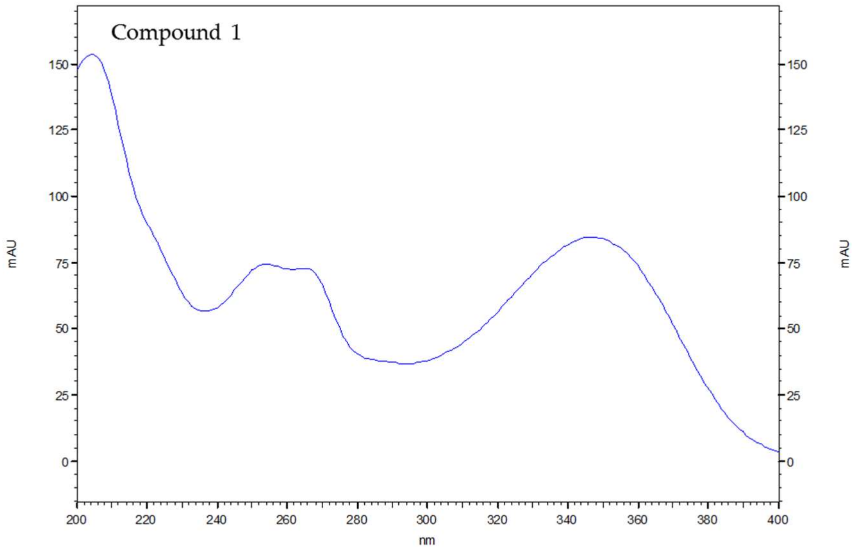

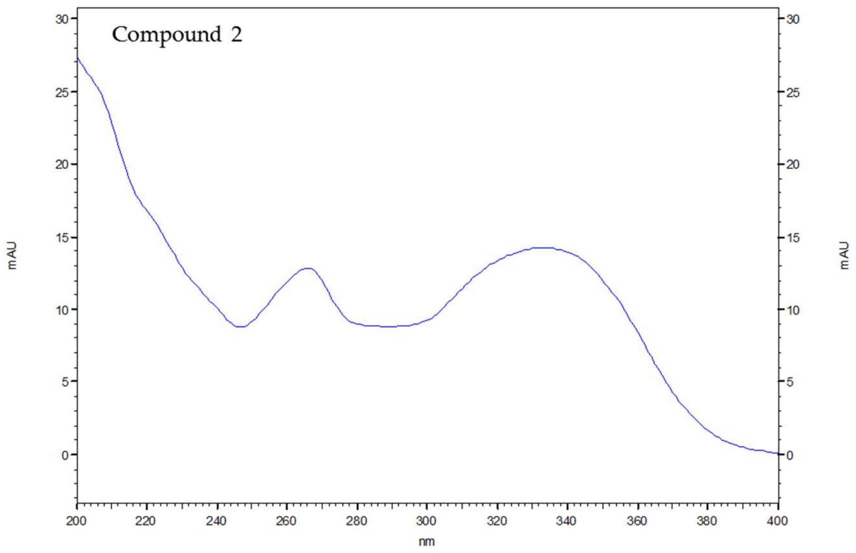
Compound 2
mAU
nm

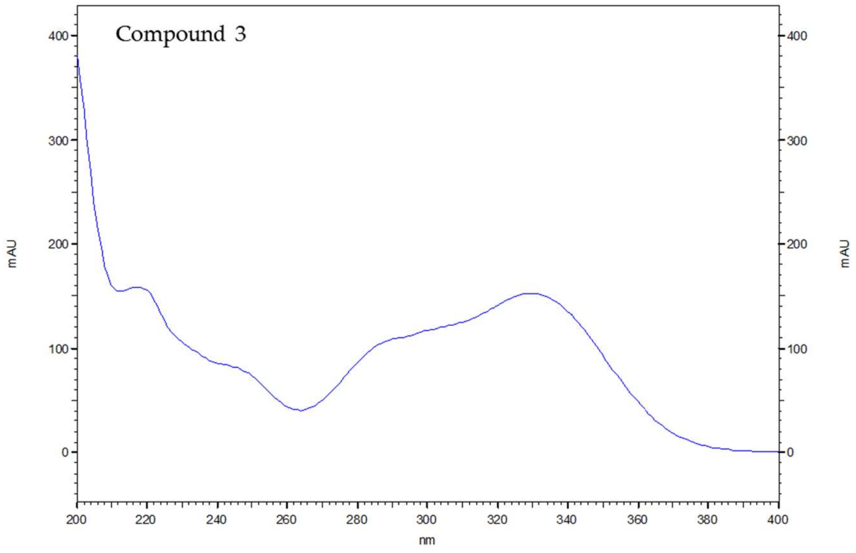
Compound 3
mAU
nm

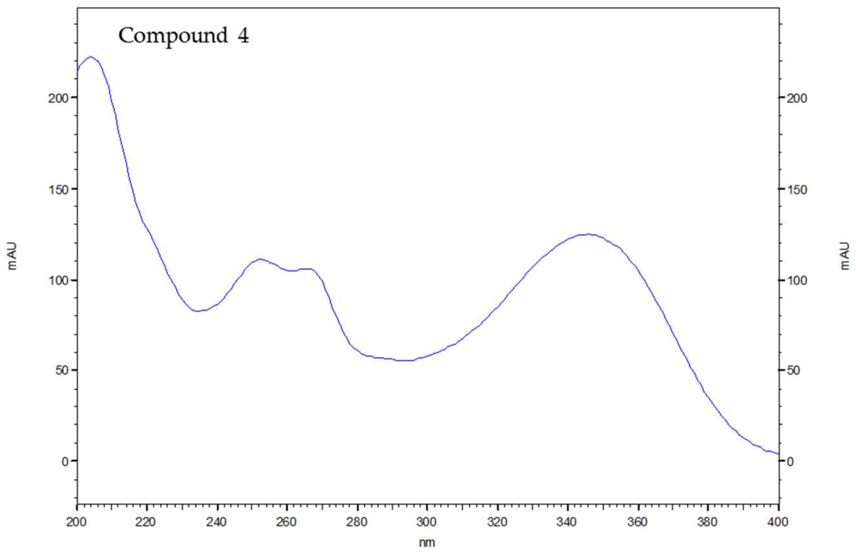
Compound 4
mAU
nm

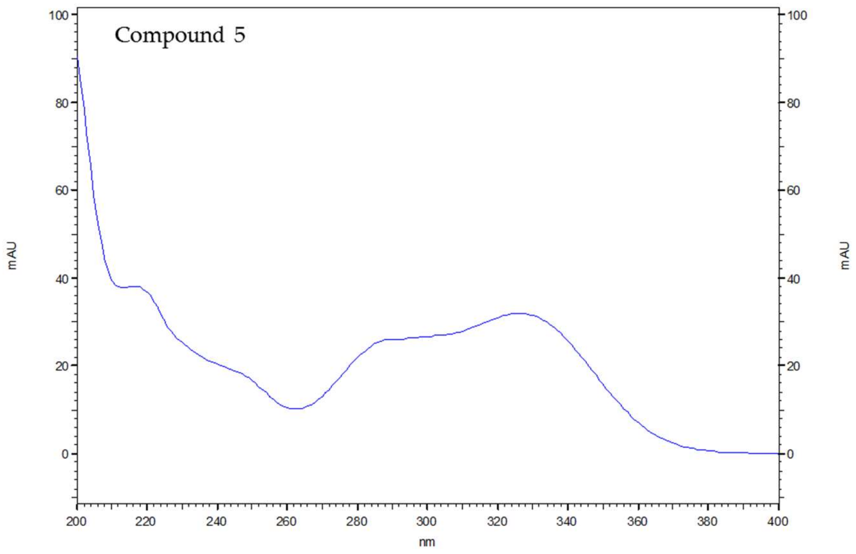
Compound 5
mAU
nm

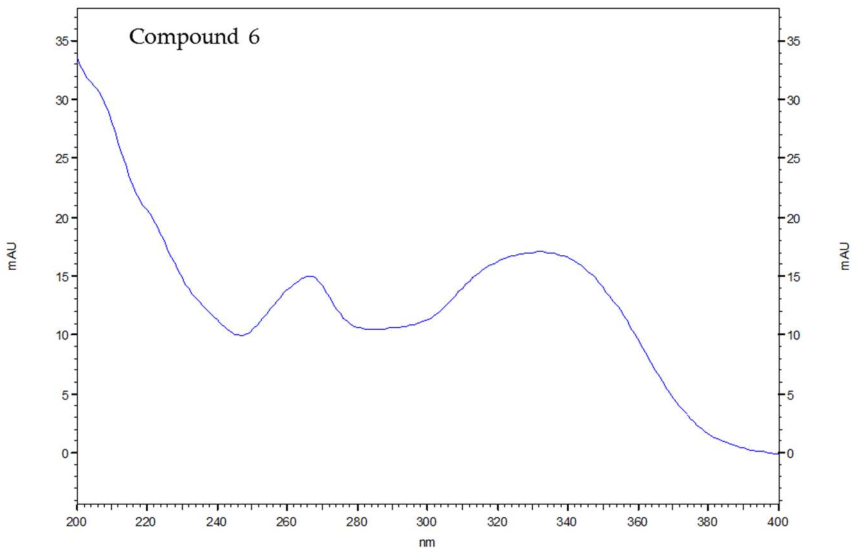
Compound 6
mAU
nm
